# Targeting Tumor Metabolism: A New Challenge to Improve Immunotherapy

**DOI:** 10.3389/fimmu.2018.00353

**Published:** 2018-02-23

**Authors:** Soumaya Kouidhi, Farhat Ben Ayed, Amel Benammar Elgaaied

**Affiliations:** ^1^Laboratory BVBGR, LR11ES31, Higher Institute of Biotechnology of Sidi Thabet (ISBST), Department of Biotechnology, University of Manouba, Sidi Thabet, Tunisia; ^2^Laboratory of Genetics, Immunology and Human Pathology, Faculty of Sciences of Tunis, Department of Biology, University Tunis El Manar, Tunis, Tunisia; ^3^Association Tunisienne de Lutte contre le Cancer (ATCC), Tunis, Tunisia

**Keywords:** T-lymphocyte metabolism, tumor cell metabolism, tumor microenvironment, immunotherapy, immune checkpoints, metabolic checkpoints

## Abstract

Currently, a marked number of clinical trials on cancer treatment have revealed the success of immunomodulatory therapies based on immune checkpoint inhibitors that activate tumor-specific T cells. However, the therapeutic efficacy of cancer immunotherapies is only restricted to a small fraction of patients. A deeper understanding of key mechanisms generating an immunosuppressive tumor microenvironment (TME) remains a major challenge for more effective antitumor immunity. There is a growing evidence that the TME supports inappropriate metabolic reprogramming that dampens T cell function, and therefore impacts the antitumor immune response and tumor progression. Notably, the immunosuppressive TME is characterized by a lack of crucial carbon sources critical for T cell function and increased inhibitory signals. Here, we summarize the basics of intrinsic and extrinsic metabolic remodeling and metabolic checkpoints underlying the competition between cancer and infiltrating immune cells for nutrients and metabolites. Intriguingly, the upregulation of tumor programmed death-L1 and cytotoxic T lymphocyte-associated antigen 4 alters the metabolic programme of T cells and drives their exhaustion. In this context, targeting both tumor and T cell metabolism can beneficially enhance or temper immunity in an inhospitable microenvironment and markedly improve the success of immunotherapies.

## Introduction

Over the past decades, huge efforts have focused on refinement of conventional cancer therapeutic strategies of chemotherapy, radiation, surgery, or targeted therapies. Although all these advances have displayed clear improvement of clinical outcomes for many types of cancers (1–3), their therapeutic efficacy remains unsatisfactory. Since the cells and the molecules of the immune system are a fundamental component of the tumor microenvironment (TME), cancer immunotherapy has emerged as a powerful new therapeutic approach to boost antitumor immunity response (4). Collectively, the immunotherapy principle consists in the modulation of the immune cells activity, predominantly T cells, using adoptive cell transfer, chimeric-antigen receptor T-cells, or monoclonal antibodies (mAbs) (5, 6). The “Checkpoint blockade” that utilizes mAbs specific to cytotoxic T lymphocyte-associated antigen 4 (CTLA-4) and the programmed cell death protein 1 pathway (PD-1/PD-L1), is arising as a newer strategy used to fight cancer and one of the most promising immunotherapies (7, 8). Indeed, encouraging results demonstrate unprecedented responses in patients with several types of metastatic tumors that were previously resistant to available treatment options (9–11). While these clinical successes have dramatically harnessed host antitumor immunity and clinical outcomes for patients, there are several limitations for immunotherapy (12). In fact, this approach is confronting a highly immunosuppressive TME and low immunogenicity of cancer cells (13). Moreover, despite the success of immunotherapy, mechanisms that govern anticancer immunity and their relevant biomarkers are still being elucidated. Therefore, the development of new methods to overcome such challenge and to improve the efficacy of this therapy is needed in cancer therapy.

Tumor-infiltrating lymphocytes (TIL) reflect tumor biology and prognostic significance. However, they are challenged with a hostile microenvironment that dampens their function and produces antitumor effects (14). Nevertheless, in the setting of malignancy, multiple mechanisms of immune suppression may exist that prevent effective antitumor immunity (15, 16). Along with negative immunologic regulators called “immune checkpoints,” TIL function is also negatively impacted by a variety of “metabolic checkpoints” (17). Increasing evidence suggests that the deregulation of energy metabolism plays a pivotal role in the inhibition of the antitumor immune response and thereby in tumor progression and metastasis (18). Under a suppressive microenvironment, TIL operate with a metabolic disadvantage since they are subjected to a lack of crucial carbon sources and increased inhibitory signals (19). This may be mainly due to the competition between T cells and tumor cells with deregulated metabolic activities, for limiting nutrients (20). Rapidly dividing tumor cells exhibit complex and dynamic metabolic reprogramming and highly glycolytic level, a phenomenon called the “Warburg effect” and recognized as one of the hallmarks of cancer (21, 22). Thus, tumor cells impede T cell access to nutrients necessary for their activation and generate high levels of lactate. The resulting nutrient scarceness and metabolic waste products accumulation in the TME lead to TIL metabolic switch that impairs their appropriate proliferation and function (23).

Collectively, the cancer cell energetics dictates the metabolic landscape of the TME. Abnormal metabolic activities of cancer cells lead to intratumoral heterogeneity and immunosuppression that could be responsible for the failure of immunotherapy (24, 25). Therefore, a deeper understanding of the metabolic challenges within the TME and their impacts on metabolic fitness of immune cells might contribute the discovery of novel promising approaches to rewire metabolic fitness of TILs that boost existing immunotherapies.

## Overlapping Metabolic Profiles of Cancer Cells and T Lymphocytes

### Metabolism Impacts T Cell Fate and Activation

T cells fate and activation is closely linked to metabolic reprogramming to acquire effector functions (26). Briefly, Naive CD4^+^ T cells can differentiate into T helper (Th) subsets or into regulatory T cells Treg, while CD8^+^ T cells differentiate into effector cytotoxic T lymphocytes (CTLs). Importantly, each T cell functional subset utilizes a distinct metabolic program (27, 28).

Highly proliferative cells increase glucose uptake and undergo upregulated aerobic glycolysis, a critical metabolic pathway for activated T cells (29). In parallel to glucose metabolism, T cell activation also enhances mitochondrial biogenesis and oxidative phosphorylation (OXPHOS) and drives mitochondrial membrane hyperpolarization, amino acid uptake, and glutaminolysis (30). There are several signaling pathways that govern the metabolic reprogramming of activated T cells. The critical checkpoint pathways known to regulate the metabolic switch are mammalian target of rapamycin (mTOR) (31) and adenosine monophosphate-activated protein (AMPK) pathways (32). The phosphoinositide-3-kinase (PI3-kinase)-Akt-mTOR pathway is a central integrator of T cell metabolism to sense and require nutrient availability in order to support high glycolytic rate in proliferating T cells (33). Notably, both activated mTOR complexes mTORC1 and mTORC2 play a role in driving glycolysis (34). Additionally, glycolysis activation is concomitant with the pentose phosphate pathway (PPP) upregulation, necessary to build-up of biochemical intermediates that are necessary for nucleotide, amino acid and fatty acid synthesis. Hypoxia-inducible factor-1α (HIF1α) is a master transcription factor enhanced by mTORC activity, which is monitoring and promoting glycolytic enzymes expression (35).

Also, in response to metabolic stress, AMPK inhibits mTOR signaling and increases catabolic metabolism (36). This results in glycolysis suppression and upregulation of oxidative metabolism and mitochondrial complex 1 activity. AMPK activation promotes generation of Treg, Th1, and Th17 subsets (37).

### Metabolism Impacts Tumor Proliferation and Progression

Cancer progression has been recognized for a long time as consequence of multiple genetic events that imply activation of oncogenes and function loss of specific tumor suppressor genes (38, 39). Increasing data point out that this is directly linked to an altered tumor metabolism. Cancer cells exhibit increased glycolysis despite the presence of oxygen, because they must divide rapidly to ensure malignant transformation and tumor development (40, 41). This phenomenon of metabolic reprogramming called “the Warburg effect,” has been recognized as one of the 10 hallmarks of cancer (42). The rate of glycolysis is largely faster than OXPHOS, providing competitive advantages to cancer cells to consume more glucose than surrounding slow-dividing cells and to grow under hypoxia and nutrient deprivation conditions over the TME (43, 44).

Furthermore, glycolysis is an effective metabolic pathway for highly proliferative cancer cells to supply nucleotide, lipid, and amino acid synthesis (45). For instance, increased levels of the glycolysis intermediates provide essential precursors for pivotal anabolic pathways such as the PPP and the serine pathway (46).

It is well established that hypoxia is as a key process supporting glycolysis in tumorigenesis (47). HIF-1α, a transcription factor induced by hypoxia, induces glucose transport by increasing expression of glucose transporters 1–3 along with the transcription of pyruvate dehydrogenase kinase (48). As a result, the tricarboxylic acid cycle is inhibited and several glycolytic enzymes activities are enhanced, including hexokinase 2 (HK2) (49) and lactate dehydrogenase A that converts pyruvate to lactate (50, 51). Therefore, intensive aerobic glycolysis generates high rate of lactate. For instance, the accumulation of lactate in TME results in acidic pH that promotes tumor progression and metastasis and contributes to cancer therapy resistance (52).

While aerobic glycolysis is considered as a key feature in cancer metabolism, clear evidence suggest that mitochondrial metabolism remains functional in most glycolytic cancer cells.

Although most cancer cells rely on aerobic glycolysis, it is clear that a tumor displays considerable heterogeneity in metabolic phenotypes (53). Such intratumorally metabolic heterogeneity may be critical for the failure of therapeutic effects. In fact, recent data has shown that cancer stem-like cells (CSCs) exhibit a distinct metabolism from the rest of tumor cells (54). This CSC metabolism depends on mitochondria function (55, 56). Moreover, the particular metabolic phenotype of CSCs may probably render them resistant to conventional antitumor therapies and explain minimal residual disease (57). Interestingly, encouraging results showed that targeting CSC metabolism (by inhibiting mitochondrial biogenesis) could be an attractive approach to reduce drug resistance (58, 59).

## T Cell Impaired Function Under Hostile TME

### Metabolic Interplay between Cancer Cells and TIL in TME

The tumor tissue consists of complex sets of cell populations including tumor cells, endothelial cells, T cells, natural killer (NK) cells, macrophages, dendritic cells, fibroblasts, and adipocytes. Regarding its genetic and metabolic diversity, this intricate network of cells contributes to the intratumoral heterogeneity. Tumors exhibit a metabolic shift and shape the TME in such a way to support cancer proliferation and metastasis (17, 60). Yet, this milieu is very hostile for T cells to mediate their antitumor effects because of hypoxia, reduced pH and acidosis, inhibitory signals, competition for nutrients, and waste products accumulation (61, 62).

It is well known that tumor cells like effector T cells (Teff), exhibit intensive aerobic glycolysis that improve their metabolic fitness and provide cell-extrinsic advantage, resulting in competition for vital metabolites such as glucose and amino acids. Therefore, tumor-infiltrating T cells are exposed to nutrient depletion in TME and become dysfunctional (62, 63). Nutrient competition has emerged as one of the major axis of tumor immunosuppression due to the anergy and exhaustion of TILs. Indeed, resources scarceness alters T cell activation and antitumor effector functions tumors through several ways (64). Rapidly dividing tumor cells impede T cell access to glucose essential for T cell metabolic switch and activation. Therefore, glucose depletion enhances AMPK pathways and decreases mTORC1 activity, glycolytic capacity, interferon-γ (IFN-γ) production, and cytolytic activity of T cells (65). This may favor Treg subsets instead of Teff and promote tumor progression. Furthermore, decreased levels of amino acids critical for efficient T cell activation and proliferative responses, can modulate the activity of TILs. Glutamine, arginine, and tryptophan deficiency in TME is immunosuppressive and dampens the proliferation of Teff subset (66).

Moreover, it has been recognized that in addition to consumption of key nutrients, tumors produce large amounts of waste products: lactate, arginine and tryptophan by-products, and phosphoenolpyruvate, that impair T cell metabolism and function and confer worse prognosis for patients (67, 68).

Lactate accumulation due to the use of aerobic glycolysis by cancer cells has been described in TME, accompanied by consequent low pH and acidification of the milieu. In mouse models, lactate levels negatively correlate with markers of T cell activation in melanoma (69). The tumor-derived lactate has positive effects on promoting survival, migration and invasion of cancer cells (70). However, lactate negatively impacts T-cell proliferation and function (71). Such acidic condition increases the expression of proangiogenic factors IL-8 and VEGF, both important involved in cancer metastasis (72). Yet, lactate inhibits the phosphatidylinositol-3 kinase (PI3K)/Akt/mTOR pathway and thus glycolytic metabolism in T cells by abolishing their cytokine production (73). Lactate also impairs the migration of T cells by reducing the chemokine receptors expression. Added to that, lactate has been demonstrated to be preferentially utilized by Tregs since they prefer oxidative metabolism, resulting in T-cell polarization toward a Treg phenotype. Excess of lactate may also regulate macrophage polarization and represses NK cells functions through a restriction of IFN-γ, IL-10, and TGF-beta (74, 75). Hence, the acidic TME has been contemplated as an attractive target for cancer therapy. Interesting results showed that buffering the tumor pH with bicarbonate improved immunotherapy outcomes.

Proliferative cancer cells create a state of tryptophan deprivation in the TME because of their increased demand for tryptophan (76). Indoleamine 2,3-dioxygenase (IDO) is a pivotal enzyme involved in tryptophan catabolism. IDO is also the first enzyme involved in the production of nicotinamid adenine nucleotide. Upregulation of IDO has been demonstrated to be correlated with an increased malignancy (77). In such context, cancer cells express high levels of IDO that deplete tryptophan availability in the TME and consequently impede T cell responses. In addition to its role in cancer cells, expression of IDO has been shown in other cells: endothelial, tumor-associated macrophages, and dendritic cells and was associated with suppression of antitumor Teff response. IDO contributes to tryptophan deprivation and degradation to kynurenine (78). Accumulation of kynurenine in TME has been described in several tumors leading to immunosuppression (79). Moreover, kynurenine is endogenously able to promote Treg cells and to reduce proliferation of Teff (40). Currently, several trials targeting IDO in combination with checkpoint inhibition are under investigation (80).

### Crosstalk between Immunologic Checkpoints and T Cell Metabolism

Immune checkpoint regulators are critical to coordinate effective and efficient immune response, to maintain self-tolerance and to prevent the onset of autoimmunity (81). Nevertheless, T cell effector function is correlated with the expression patterns of coinhibitory and costimulatory immune checkpoint receptors (82). The most described checkpoint proteins playing a central role in maintain immune self-tolerance belong to the TNFR superfamily (83) and B7 family (84).

Tumors can evade immune surveillance through defective immune-checkpoint signaling pathways (81, 85). It is now clear that under tumoral context, aberrantly expressed inhibitory checkpoint proteins are described to disrupt antitumor immune response. CTLA-4 and PD1 are critical coinhibitory receptors highly expressed in T cells under TME (86). Moreover, PD-1 ligands PD-L1 and PD-L2 are upregulated by cancer cells and thus disrupt T cells mediated antitumor response (87). Accordingly, immune checkpoints ligand–receptor interactions were proven to be effective targets to enhance antitumor immunity moving immunotherapy into a new era (88). In fact, immune checkpoints blocking antibodies have achieved an outstanding benefit in cancer treatment enabling patients to produce an effective and durable antitumor response. Currently, three checkpoint inhibitors are approved for the treatment of advanced melanomas: ipilimumab, a CTLA-4-specific mAb (89), and pembrolizumab and nivolumab, which are PD-1-specific mAbs (11). Furthermore, remarkable clinical effectiveness has been reported in other cancers such as, ovarian (90) non–small cell lung carcinoma (91), breast (92), prostate (93), and lymphoma (94).

Although the effectiveness of the immune checkpoint blockade in enhancing antitumor immunity by reducing the number and/or the suppressive activity of Tregs and by restoring the activity of Teff has been reported, little is known about mechanisms underlying T-cell activation. Recent evidence suggest that both checkpoint ligation and inhibition may directly modify metabolism of T cells and cancer cells and alter their metabolic feature. Emerging data have shown that PD-1 binding to its ligands impairs the metabolic phenotype of TIL, by inhibiting glycolysis and upregulating fatty acid oxidation (FAO) (95, 96). CTLA-4 ligation to B7 inhibits glycolysis without augmenting FAO, which suggests that CTLA-4 would not affect the metabolic profile of non-stimulated cells (95). Hence, this abrogation of energy generation impacts antitumor response and leads to reduced cytokine secretion and Teff exhaustion (97). Moreover, immune checkpoints also have an impact on cancer cell metabolic reprogramming. Ligation of PD-L1 directly upregulate glycolysis in cancer cells by promoting glucose uptake and production of lactate (98). Hence, signaling through PD-L1benefits cancer cell metabolism, leading to their expansion and survival (61).

Interestingly, the immune checkpoint blockade appears to differentially impact the metabolic profile in TME by favoring T cell activation and in contrast inhibiting cancer cells. Blocking PD-1 and PD-L1 may reduce glycolysis level in cancer cells by inhibiting mTOR pathway (61). Consequently cancer glucose uptake and lactate secretion decrease which restore glucose availability in TME. Besides, the immune checkpoint blockade has a benefit on T cell metabolism and function. A melanoma mice model study showed that tumor treatment with immune checkpoint inhibitors increases glucose rates in TME and enhances T-cell glycolysis and cytotoxic function (99).

In conclusion, clear evidences demonstrated that tumor cell metabolism deeply affects TME differentiation and functions. By modulating tumor cell metabolism, one can control nutrient availability for T cells, thus promoting either their antitumor or immunosuppressive functions.

## Targeting Metabolism for Efficient Immunotherapy

### Targeting Glucose Metabolism

In tumors, T cell activation and proliferation could be impaired by metabolic disruption, therefore cell metabolism becomes an attractive target to restore anti tumor immunity and to develop anticancer therapy (100). However, in tumoral context, it is wise to consider the overlapping metabolic requirements of tumor and immune cells.

Several drugs have been proposed to target tumor glucose metabolism for cancer treatment. For instance, inhibition of glycolytic enzymes that catalyze several steps of glucose metabolism has been known to support anticancer effects (101). 2-Deoxyglucose (2DG) is a non-metabolizable glucose analog and inhibitor of HK used to shut down glycolysis since the first steps. Despite the safety of this drug in cancer patients and its efficiency beyond glycolysis inhibition in cancer cells (102–104), 2DG has also been shown to impair the metabolism of T cells, which results in decreased secretion of cytokines and reduced T cell antitumor function that may be critical for therapeutic success (105). Dichloroacetate (DCA) is another drug targeting cancer cell metabolism which showed conflicting results. DCA is a metabolic disruptor inducing a shift from glycolysis to OXPHOS and inhibiting growth of tumor cells *in vitro* (106, 107) and in mouse models (108). Similar to 2DG, DCA is not specific to tumor cell metabolism, therefore, it mediates the same metabolic shift in T cells, favoring Treg formation (109).

The TME is particularly immunosuppressive because of lactic acid production in the extracellular milieu that may stand against the therapeutic efficacy (110). To overcome the “Warburg effect” in cancer cells, some therapeutic approaches target lactate with lactate dehydrogenase (LDH) and monocarboxylate transporter (MCT) inhibitors or oral bicarbonate supplementation to tamper the acidic microenvironment (111). Importantly, the inhibition of LDH, the enzyme that catalyzes the conversion of pyruvate into lactate, shows impaired glycolysis and growth arrest in cancer cells (51, 112). Moreover, lactate blockade improves the response to 5-fluorouracil treatment in colorectal cancer (113). However, LDH inhibition demonstrates contradictory results in proliferating T cells response. While it has been reported that deletion of LDH using small-molecule FX11 or Galloflavin ameliorates lactate levels (114, 115), other studies demonstrate that such inhibition leads to a decrease in T cells IFN-γ production (116). Therefore, the differential impact of LDH inhibitors on cancer and immune cells should be considered when administrated for tumor therapy.

Beside the inhibition of the enzyme LDH, the lactate transporters MCT-1–4 may also be targeted to avoid acidic milieu (117). MCT of the *SLC16A* gene family influences substrate availability, the metabolic path of lactate and pH balance within the tumor (118). Recent studies have described new MCT disruptors, thalidomide, lenalidomide, and pomalidomide that act on cancer cells to impair the CD147–MCT-1 ligation (119, 120). In addition, the treatment with lenalidomide has been reported to enhance IL-2 and IFN-γ secretion in T cells (121), suggesting that lenalidomide could suppress tumor cell proliferation while favoring T cells activation. Although these drugs cause a loss of cell surface expression of MCT-1, the efficacy may be limited as cancer cells express not only MCT-1 but also MCT-4. Further, AZD3965 another lactate transporter inhibitor, is currently in phase I clinical trials for advanced solid tumors and diffuse large B cell lymphomas (http://www.clinicaltrials.gov/ct2/show/NCT01791595). AZD3965 is targeting MCT-1/MCT-2. Yet, the inhibitory effect has also been observed in T cells (122). Recently, the effect of diclofenac, a non-steroidal anti-inflammatory drug, has been investigated on lactate transport and secretion. Diclofenac has been reported to reduce tumor growth, the number of infiltrating Tregs and the lactate rate in the microenvironment in glioma model (123, 124). Therefore, this result raises the possibility that the application of diclofenac should be feasible to improve the efficacy of immunotherapies.

Further, lactic acid production and resulting low-pH TME are shown to dampen CTLs proliferation and cytotoxic response (125–127). Hence, neutralization of TME may have a meaningful impact on improving the efficacy and outcomes of anticancer immunotherapy therapeutics (128). Emerging data show that buffering lactic acid with bicarbonate or proton pump inhibitor, Esomeprazole improves the pH of TME (129, 130). More importantly, neutralization of TME pH improves outcomes in CTLs and in NK cell mediated anticancer as well. Notably, buffering TME with oral bicarbonate inhibits tumor growth when combined with anti-PD-1 immunotherapy in a melanoma model, and improves survival when combined with adoptive T-cell transfer (131). Altogether, these data indicate that targeting TME acidification by buffering provide a new perspective for immunotherapy outcomes.

The PI3K-AKT-mTOR is an important pathway well known to play a critical role in cancer and immune cell metabolism (31, 132). Further, this pathway has been extensively studied in various cancers showing inappropriate activation supporting tumor growth and survival. Over the last decades, several therapies were developed against mTOR signaling in several solid malignancies (133, 134). Analogs of rapamycin, a drug that inhibits the mTOR signaling, have been approved for the treatment of breast (135), renal (136), and pancreatic cancers (137). An increasing number of studies have reported that inhibition of the mTOR pathway suppresses the glycolytic metabolism and sensitizes tumor cells to chemotherapy (138, 139). Yet, it has been reported that rapamycin can mediate opposite effects on T cells since it broadens Tregs and cytotoxic memory T cells but at the same time decreases Teff proliferation (140). Interestingly, recent evidence suggest that treatment with rapamycin combined with immunotherapy augments cytotoxic and memory T-cell functions in glioblastoma cancer (141). Therefore, rapamycin could be an attractive adjuvant to be used in combination with immunotherapy.

Besides glycolysis, OXPHOS is also a possible target structure in cancer cells. Several reports have described the potential effects of metformin, which is commonly used to treat type II diabetes, as an anticancer drug. Indeed, a large number of retrospective clinical studies and randomized control trials show that metformin prevents tumor growth and improves clinical prognosis in various cancers including lung and prostate cancers (142, 143).

Interestingly, those effects seem to be partially immune-mediated as metformin improved T cell function *in vivo* (144). Further, metformin has been proposed as a treatment for melanomas due to the limitations of current therapies (145). Metformin is known to target the mitochondrial respiratory complex I and to activate AMPK pathway signal transduction (146, 147). Several reports have demonstrated that AMPK plays pleiotropic and conflicting effects at the interface of cellular metabolism and function (37). In fact, activated AMPK may engender both antitumor and protumor effects in a manner not yet understood (148, 149). Notably, activated AMPK pathway impedes mTOR signaling, and shuts down glycolytic gene expression leading to antiproliferative effects in cancer (150, 151). However, AMPK activation on another side helps cancer cells accommodation to metabolic stresses, which raises their survival (152). Metformin’s AMPK activating effects could also impact T cells behavior mainly by enhancing memory T cells (105, 153) and Treg expansion (154). Therefore, metformin treatment may improve secondary responses. Yet it could favor immunosuppressive Treg cells in TME.

### Targeting Amino Acid Catabolism

In the context of the TME, cancer cells require a continuous and high rate of supply of energy to take advantage of their metabolic reprogramming and to avoid immune surveillance. In fact, cancer cells create a state of nutrient deprivation for the T cells and redirect glucose and amino acids for their own advantage. It is well known that l-arginine, tryptophan and glutamine are fundamental in tumor progression and immunity (155). Therefore, targeting theses amino acids in cancer therapy becomes a promising strategy for the development of novel therapeutic agents (156). In fact, many clinical trials are actually testing specific drugs inhibiting amino acid metabolism in cancer cells. Depletion of arginine was assessed using ADI-PEG20 inhibitor (157). It can inhibit cell proliferation *in vitro* and tumor growth *in vivo and* decrease Treg accumulation (158). However, it would be more pertinent to prevent amino acid depletion by tumor cells or myeloid cells rather than decreasing amino acid rates in the TME. This approach is currently tested in a clinical trial with CB-1158, an ARG inhibitor, in combination with checkpoint therapy (159).

Furthermore, increasing evidence suggest that tryptophan is critical in supporting oncogenic signature and in maintaining the immunosuppressive phenotype in several cancers (160). Interestingly, it has been reported that the silencing of IDO boosted antitumor immunity in metastatic liver tumor model (161), improved cytotoxic T cell function and decreased Treg numbers (162). Accordingly, it is well established that IDO is a key target of drug discovery in cancer immunotherapy (80, 163). Imatinib is another drug displaying improved anti tumor immunity by activating T effector cells and suppressing Tregs, in a manner dependent on IDO pathway (164). For instance, a current clinical trial is assessing the combination between imatinib and anti-CTLA4 approach in GIST (165).

Glutamine is considered as a critical amino acid for cancer cell metabolism as well as for rapidly dividing T cells. To overcome the high glutamine consumption rates of cancer cells, several therapeutic agents targeting glutamine metabolism have been explored in preclinical studies (166). Three compounds were assessed as glutamine analogs, 6-diazo-5-oxo-L-norleucine, azaserine, and acivicin. These agents showed impaired activity of enzymes utilizing glutamine in many tumor models (167, 168). Moreover, testing glutamine transporter inhibitors gamma-l-glutamyl-*p*-nitroanilide and benzylserine [H-Ser(Bzl)-OH], showed reduced glutamine uptake and cell growth in lung and prostate cancers (169, 170). Yet, glutamine plays also a key role in normal Teff. Therefore, it is conceivable to consider better tumor-targeting options under the TME.

## Concluding Remarks

**Figure 1:**
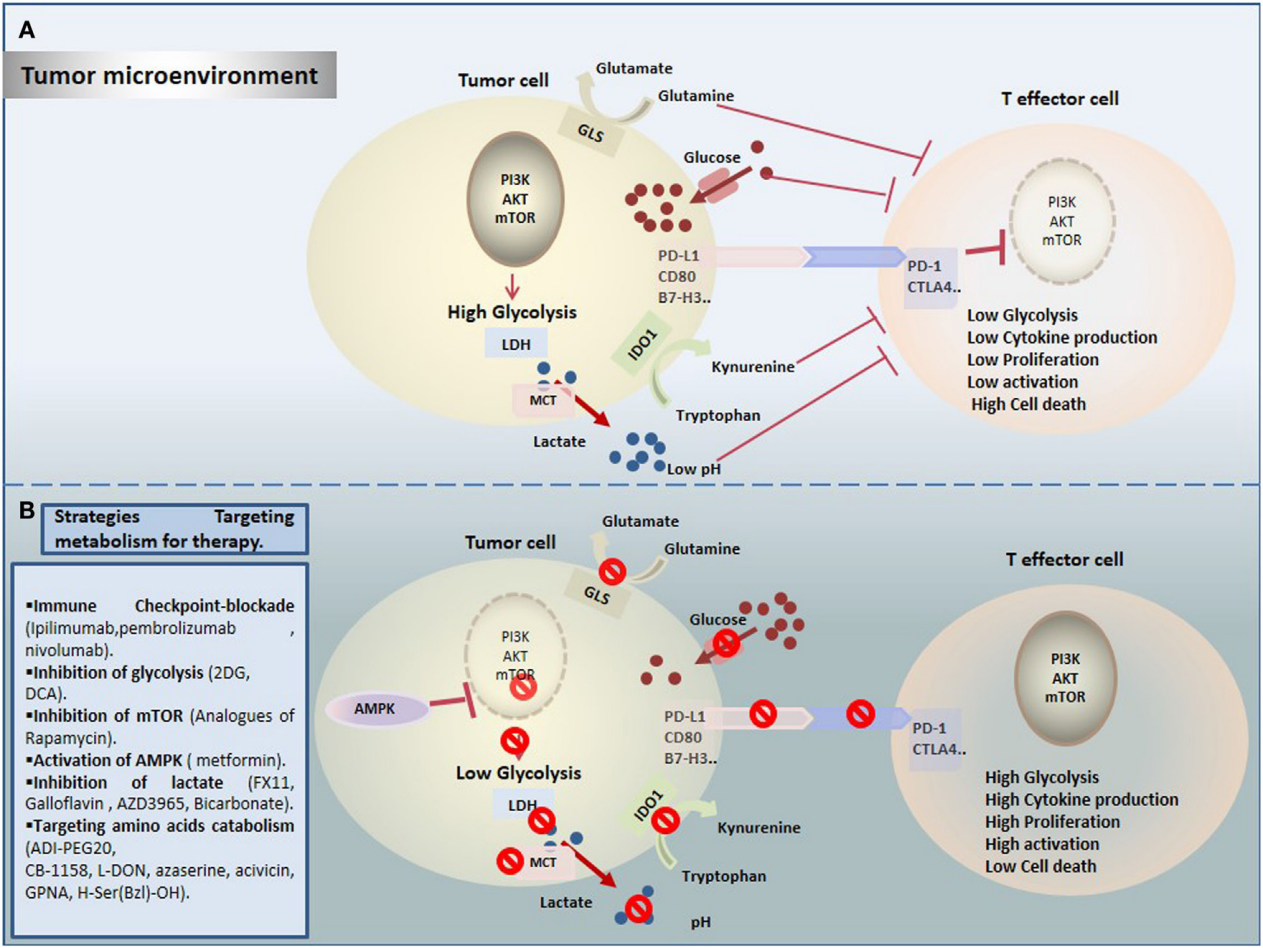
Therapeutic targeting cell metabolism in the tumor microenvironment (TME). **(A)** Tumor cells create a hostile TME that affects metabolic fitness of T cells through multiple ways. T cells are challenged by different immunologic and metabolic checkpoints: Glucose and amino acid depletion, high acidity and lactate, and upregulation of immune checkpoints influence T cell metabolism to suppress glycolysis thereby reducing their activation and proliferation. **(B)** Currently, several novel promising approaches are proposed to rewire metabolic fitness of T cells in the TME and to boost existing immunotherapies.

Cancer immunotherapy provides successful and powerful opportunity in cancer treatment. However, it is important to get comprehensive understanding of mechanisms leading to reduced antitumor immunity under hostile TME. Importantly, TILs have to surpass not only immune checkpoints but also a wide range of metabolic checkpoints that fate their energetic behavior defects and dampen their function. In fact, cancer cells upregulate nutrients uptake and waste metabolites production to generate an immunosuppressive TME that allows their evasion and growth, and that dictates immune cell fate (Figure 1A). Increasing emerging data point out the modulation of cellular metabolism, using combinational approaches of metabolic disruptors with immune checkpoint blockade (Figure 1B). However, a special attention should be devoted to target specific tumor site, in order to avoid systemic toxicity and innumerable other side effects. In summary, by operating through distinct and complementary mechanisms, these new therapeutic strategies might reinvigorate TILs by restoring their metabolic properties and improving the efficacy of immunotherapies.

## Author Contributions

All authors listed have made a substantial, direct, and intellectual contribution to the work and approved it for publication.

## Conflict of Interest Statement

The authors declare that the research was conducted in the absence of any commercial or financial relationships that could be construed as a potential conflict of interest.

## Abbreviations

2DG, 2-deoxyglucose; ACT, adoptive cell transfer; AMPK, AMP-activated protein kinase; ATP, adenosine triphosphate; CAR, chimeric-antigen receptor; CTLs, cytolytic T cells; CTLA-4, cytotoxic T-lymphocyte antigen; FAO, fatty acid oxidation; HIF, hypoxia-inducible factor; IDO, indoleamine 2,3-dioxygenase; IFN-γ, interferon-γ; LDH, lactate dehydrogenase; MCT, monocarboxylate transporter; mTOR, mammalian target of rapamycin; OXPHOS, oxidative phosphorylation; PD-1, programmed death-1; PD-L1, programmed death ligand-1; PI3K, phosphatidylinositol-3 kinase; PPP, pentose phosphate pathway; TCA, tricarboxylic acid; Teff, effector T cells; Th, helper T cells; TIL, tumor-infiltrating lymphocytes; TME, Tumor microenvironment; Treg, regulatory T cells.
